# How do older adults understand and manage distress? A qualitative study

**DOI:** 10.1186/s12875-020-01152-7

**Published:** 2020-05-04

**Authors:** Alice Moult, Tom Kingstone, Carolyn A. Chew-Graham

**Affiliations:** 1 Keele Medical School, Keele University, Staffordshire, ST5 5BG UK; 2 School of Primary, Community and Social Care, Keele University, Staffordshire, ST5 5BG UK; 3 Midlands Partnership NHS Foundation Trust, Stafford, ST16 3SR UK; 4 Applied Research Collaboration (ARC) West Midlands, London, UK

**Keywords:** Older adults, Distress, Self-management, General practitioner, Primary care, Mental health

## Abstract

**Background:**

Distress is an expected emotional response to a negative life event. Experiences common in later life may trigger distress such as bereavement or loss of physical mobility. Distress is considered to be distinct to anxiety and/or depression and is not diagnostically labelled as a mental health problem. Older adults will often manage their own distress. Previous literature has focused on how younger adults self-manage mental health problems, however little research has explored the self-management strategies used by older people. There is a need to clarify the role of primary care in the context of distressed older adults who may consult healthcare services. This study seeks to address these gaps through qualitative methods.

**Methods:**

Keele University’s ethical review panel approved this study. We recruited older adults who self-identified as distressed from community groups in North Staffordshire, England. Data were generated through semi-structured interviews and analysed thematically using constant comparison methods. A patient and public involvement and engagement group contributed to development of the research questions and methods, and offered their perspectives on the findings.

**Results:**

After 18 interviews data saturation was achieved. Key themes were: experiences of distress, actions taken, help-seeking from healthcare services and perceptions of treatments offered in primary care. Various forms of loss contributed to participants’ distress. Participants initiated their own self-management strategies which included: pursuing independent activities, seeking social support and attending community groups and church. Five participants reported having consulted a GP when distressed but described a lack of acceptable treatments offered.

**Conclusions:**

To support older adults who are distressed, healthcare professionals in primary care should consider exploring how patients currently manage their mood problems, provide a broad range of information about potential management options and consider sign-posting older adults to community resources.

## Background

Experiences of distress are prevalent in older adults [1]. Distress is a subjective experience [2] which may be triggered by a range of social factors or life events such as bereavement or receiving a diagnosis of a physical health problem [3, 4]. Distress could be related to depression and anxiety, but is also distinct [5]. The term distress is not a diagnostic label, but a means of representing an individual’s sense of their lived experience. In contrast, both depression and anxiety are used as psychiatric labels within the Diagnostic and Statistical Manual of Mental Disorders Fifth Addition (DSM V) [6] and the International Classification of Diseases Tenth Addition (ICD-10) [7]. Geraghty et al. [5] suggested that an individual reaching the diagnostic criteria for depression and anxiety are likely to have expressed feelings of distress. However, the reverse may not be the case, an individual experiencing distress may not meet the diagnostic criteria which would infer a diagnosis of depression or anxiety. Along with being diagnostic labels, the terms depression and anxiety are part of Western societies’ every-day vocabulary, they represent ‘idioms of distress’ which describe an individual’s mood state [8]; their widespread use has blurred the boundary between distress and mental health problems. According to Thangadurai and Jacob [9], a biomedical approach has been inappropriately applied to everyday stressors in life. The medicalisation of distress has resulted in General Practitioners (GPs) focusing on bio-medically treating individuals for mental health problems and, resultantly, ignoring the impact of social and economic stressors [9].

Most older people manage their feelings of distress on their own and do not seek help from healthcare services [10]. This could be because older adults do not recognise distress as a biomedical problem and do not view biomedical treatments as appropriate [11]. Through exploring lay perspectives on the solutions offered by GPs to patients experiencing distress, Geraghty et al. [12] reported that GPs primarily offered reassurance, time off work or medication to help them to sleep. Geraghty et al. also suggested that GPs sometimes labelled patients as ‘depressed’ to access a wider range of treatments such as antidepressants or psychological therapies. Interventions are needed which de-medicalise distress and point towards self-management strategies.

Self-management is defined as ‘taking increased responsibility for one’s own health, behaviour and well-being’ [13]. Effective self-management encompasses the ability to monitor one’s own health problems and to have the cognitive, behavioural and emotional responses necessary to maintain a satisfactory quality of life [14]. Whilst policy initiatives support GPs to encourage older adults to self-manage both physical and mental health problems [15, 16], this relies on the assumption that older people can effectively self-manage their health. There may be some older adults who cannot effectively self-manage their distress and these individuals may seek guidance from a GP. There is a need to clarify the role of primary care for those few older adults experiencing mood problems who find insufficient solutions from their own actions. In clarifying these roles, GPs may be able to provide appropriate support to older adults who are likely to experience distress due to negative life events. Supporting these older adults experiencing distress may prevent their mood problems progressing into mental health disorders [17].

Whilst research has explored how younger adults self-manage depression and found that a number of strategies are used (e.g. exercising, socialising with friends and internet use) [18, 19], previous research has not explored how older people self-manage mood problems. The aim of this study is to identify the self-management strategies distressed older adults employ. This could be useful information as healthcare professionals could become more aware of their role in supporting distressed older adults. Healthcare professionals may gain an understanding of how distressed older adults try to support themselves and, if these self-management strategies have been unsuccessful, try to find alternative solutions which may alleviate the older adult’s distress.

## Methods

### Study design

To explore how older adults self-manage distress a qualitative approach was adopted. Keele University’s ethical review panel approved the study (ERP1279; 16.9.2016). All participants provided written consent prior to data collection.

### Patient and public involvement and engagement

Patient and public involvement and engagement (PPIE) is the act of doing research ‘with’ the public, rather than ‘to’ the public’ [20]. A PPIE group, convened of older adults with mood problems, advised this study. PPIE members suggested that distress was prevalent in older adults and that older people should take more responsibility for their health, making self-management a suitable research focus. We sought PPIE advice when developing the research questions and methods, and when analysing the data. Modifying the research questions ensured relevant questions were being addressed. Developing the methods with a PPIE group ensured that public facing documents, the topic guide and ‘think aloud’ activities were appropriate for the target audience and gaining PPIE members’ perspective of the data could have arguably enhanced the trustworthiness of the interpretation.

### Sampling strategy and recruitment

Older adults who self-identified as distressed were recruited through existing community groups in North Staffordshire, England. As older adults may not perceive distress as a biomedical problem [11], recruiting from community groups provided access to older people who may not have presented their distress within primary care settings.

To ensure the sample consisted of a mixture of participants from various demographic backgrounds, a purposive sampling [21] strategy was employed. Inclusion criteria were used to ensure the sample consisted of individuals who were 65 years or older and who self-identified as distressed; we employed a similar strategy in previously published research [22]. Distress was described as feeling ‘low’ or ‘stressed’ on the information leaflets. The information sheet also described that participants had to currently self-identify as distressed, or have been through an experience of distress within the past 12 months. The authors also tried to recruit older adults who did not attend community groups through snowball sampling techniques [23]. Community group attendees were given information leaflets and were asked to distribute them onto friends who might be interested in participating in the study; this was not fruitful.

### Data generation

Data collection took place between September 2016 and March 2017. Interviews lasted between 44 and 92 min (mean 63 min). Although given a choice of a preferred venue in which to be interviewed (within the community group building or at home), each participant chose to be interviewed within a private room at the building where the community groups were held. Semi-structured interviews were used as they were sufficiently structured to address dimensions of the research question, but also permitted participants to offer new meanings to the topic under study [24]. Whilst data collection was on-going, the authors met regularly to discuss the data, and the original topic guide (see Additional File 1) was refined as recruitment continued.

Interviews were digitally recorded and transcribed. Data was anonymised and each participant was given a pseudonym. Data was analysed thematically [25]^,^ drawing on methods of constant comparison [26]. This method of analysis was inductive with codes derived from the data; these were refined and grouped to develop themes within and between transcripts. Data analysis was conducted by three researchers from various disciplinary backgrounds. Interpretations and emerging themes were discussed as a team until a consensus was achieved. To increase the trustworthiness of the interpretation [27], PPIE members were shown quotes which illuminated a theme or sub-theme. PPIE members’ interpretations of the data did not differ from the researchers’. Recruitment ceased once data saturation, defined as when no new codes or themes were identified in the data [28], was reached. NVivo 10 was used to facilitate the analytic process.

## Results

### Sample characteristics

The sample was comprised of 18 older adults (11 females, 7 males) with a mean age of 77.5 years (range 65–91 years). Whilst ethnicity was not a criterion for inclusion, all participants were White British; this reflected the demographic characteristics of the community groups we recruited from. Limitations of the sample are considered in the discussion.

The following themes will be presented: experiences of distress, actions taken, help-seeking from healthcare services and perceptions of treatments offered in primary care. Illustrative data are presented to support interpretation and are identified by participant pseudonyms.

### Experiences of distress

Within this theme two subthemes will be presented which detail the language that participants used to describe their distress, and the reported causes of distress.

### Language of distress

Participants used a variety of terms to describe their distress, including feeling: “*low*”, “*stressed*”, “*worried*”, “*run down*”, “*off*” or “*angry*”. Participants often used terms in combination to conceptualise their distress. Five participants sought help from healthcare services and used language informed by contact with these services. Three participants disclosed seeking help from a GP when distressed and reported receiving a label of depression, these participants used terms such as “*depressed*” to describe their mood problems. A further two participants consulted a GP and reported receiving a label of anxiety:

*“I went to my doctors and he said I’d got anxiety. You know … I'd be saying to friends that had lost someone dear to them, ‘I am sorry and I am sorry’ but I didn't know the depths of it until it happened to me and I think that is the same with this anxiety.”*

(Diane)

When discussing their mood problems, the terms “*depressed*” or “*anxious*” were only used by participants who had received a label of anxiety or depression from a GP.

### Perceived causes of distress

All participants reported attributing their distress to some form of loss, one being the loss of a job, through retirement:

*“After retirement and at first when I retired, because I enjoyed the job meeting them many people, it was hard, it was difficult, yeah, as I say you knew all the [people] they knew you and they just let you get on with your job, and obviously you had to be certain places like when taking the meals to the [people] and stuff and you had to be a certain places at certain times, but the rest of the time, yeah, it was a complete pleasure.”*

(Leslie)

Most retired participants reflected positively on their previous employment as it had provided an opportunity for them to socialise with other employees and members of the public, retirement removed this opportunity for social contact.

Sixteen participants disclosed suffering from physical health problems, as described in Table 1. Some participants reported that physical health problems had caused a loss of physical mobility which resulted in them becoming isolated within their homes:

*“I have recently err last year, had a fracture on my hip and the err, fractured the femur so I was in the house for ... from October to March without going out.”*

(Anne)

After undergoing hip surgery, Anne discussed how her friends struggled to visit her in her home, which led to social isolation. Participants also attributed experiences of distress to a spouses’ loss of physical mobility. These participants reported taking on a caring role for their spouses to ensure their basic needs were met (e.g. having food to eat). One participant, Helen, described that she “*had to*” care for her husband, suggesting this role was obligatory.

**Table 1:** Participant demographic and health information

Pseudonym	Retired	Married Partner	Self-reported physical health problems
Anne	Yes	No	Diabetes, osteoporosis, recovering from hip replacement
Barbara	Yes	Yes	Stroke
Carol	Yes	No	Breast cancer (in remission), recovering from hip replacement, broken wrist and rib, bladder problems
Diane	Yes	Yes	Recovering from hip replacement, actinic keratosis
Elizabeth	Yes	No	Arthritis, COPD, recovering from hip replacement
Frances	Yes	Yes	Arthritis, high blood pressure
Gillian	Yes	Yes	Heart murmur, stroke
Helen	Yes	Yes	Sleep apnoea, benign brain tumour
Irene	No	Yes	None
Janet	Yes	Yes	Arthritis, COPD
Kathleen	Yes	Yes	Burst ear drum, awaiting small bowel resection
Leslie	Yes	Yes	Broken ankle, high cholesterol
Michael	Yes	Yes	Minor hearing loss, glaucoma
Nigel	Yes	Yes	Recovering from heart surgery
Owen	Yes	Yes	Diabetes, high cholesterol
Peter	Yes	No	None
Robert	No	No	Arthritis, COPD, awaiting shoulder and spinal surgery
Stephen	Yes	Yes	COPD, recovering from heart operation

*COPD Chronic Obstructive Pulmonary Disease

A few participants attributed their experiences of distress to grief and reported feeling alone. Diane’s husband died over 20 years previously and she still described feelings of loneliness:

*“I start dulling my mind and not being interested, I don't know, so I think keeping interests helps with loneliness but you can still be lonely at times, I still miss my husband.”*

(Diane)

Some participants described that the loss of multiple friends added to their feelings of loneliness and distress:

*“I went to erm two funerals recently and somebody said ‘are you alright [Anne]?’ to me when I was at the second one, and I said not really because I had a phone call just before I came out to say another friend had died.”*

(Anne)

With the loss of each friend, participants reported having to deal with an accumulation of grief and the loss of social contacts.

### Taking action

Once participants had recognised that they were experiencing distress, they each described taking some form of action. Most of the participants identified their own ways of managing distress, which did not involve healthcare services. Attitudes of stoicism were presented towards mood problems, as described by Elizabeth when discussing how she managed her distress (attributed to a hip replacement she had 1 year prior to the interview):

*“Erm so I had to sort of wash myself, strip washes and things because you weren't allowed in the bath erm … it was stressful but I just had to get on with it, I just had to get on, I think it is because all of my life I've had to work, I've never had anything given to me on a plate ... erm ... erm, I brought my children up, my first husband left and left me with two teenagers, which was a struggle, but I managed and I married again and I was married for over twenty years and he died eight years ago. But you know what? I just got on with it, so that is what I do now.”*

(Elizabeth)

Participants who reported experiencing previous challenges in their lives described how such experiences supported their own sense of resilience and self-sufficiency; they applied this to the way they managed their distress.

To manage their distress, participants pursued independent activities such as reading, gardening and walking. These activities provided positive source of distraction:

*“Oh yes, I'm an avid reader, when I go to bed at night I cannot go to sleep unless I have read, sometimes I've been known to, if it is a good book, to read all night, if it is a good book, it does help because you've got your mind on the book instead of on the things that are worrying you because you put yourself in the situation that you're in in the book.”*

(Gillian)

*“I just see [the garden] as a place where I can go out and forget about everything and I do (laughs) yeah it takes time and effort and it is an on-going job, but I like taking the cuttings and everything about it.”*

(Helen)

*“The walks, erm well they keep me going mentally and physically, erm if I, if I stayed in I don't think that would be good, I think the mind would start to play tricks and I think I'd get problems but I go out, I have to go out.”*

(Peter)

Activities such as gardening were helpful as they provided an ongoing task; a continual source of distraction from feelings of distress. Gardening and walking also helped to get people out of the house. Some participants, however, described that a lack of physical mobility presented a barrier to gardening:

*“My garden is beginning to look a bit untidy and it erm, it is getting at me, so when I go out I start pulling weeds and what not, then my back starts so I best be careful.”*

(Gillian)

Ten participants identified as either Catholic or Christian and described that practising their religious beliefs provided them with a sense of inner-strength, as described by Diane:

*Diane: “Yes, yes, I am a Roman Catholic.”*

*Interviewer: “How does that help?”*

*Diane: “It equips you for when the storm hits you, somehow, I don't know what it is, you don't avoid that storm but somehow you're not alone in it and, you know someone is there, and it provides inner strength.”*

The perception that God was present within their lives prevented some participants from feeling like they were facing their distress alone. A few participants, who held religious beliefs, also described attending church as it provided a source of social contact. Attending church facilitated friendships between participants and members of their congregation.

Many participants, who did and did not attend church, sought social support from their friends to self-manage their distress. Female participants particularly reported seeking social support from their female friends:

*“Call my friend [Helen] who I'm here with today and erm we talk a lot, we talk a lot on the phone, erm and sometimes I shall say right I'm going see [Helen] and my husband will say ‘oh I'll come with you’ and I say, ‘no you’re not it is going to be a ladies’ afternoon’.”*

(Irene)

Female participants expressed a preference to discuss their problems with female friends, rather than their spouses. Although participants felt that their family members were a constant presence in their lives, some were concerned about discussing factors which contributed to their distress with members of their family. Carol discussed how she told her sister that she was suffering from a physical health problem:

*“I was apprehensive, erm my sister was saying ‘why are you going to the hospital?’ and I said, ‘oh I've got a cyst’ and she was saying ‘are you sure it is a cyst, are you sure? What have they told you? Have they done this and that?’ and I said, ‘yeah they've done everything there is to do and it is alright, don't worry’, oh she was on the phone at me, so I thought, no, it is alright, it is alright, it is alright, you know and then I didn't want her to know because I knew she'd fall apart, so I had to write it all down in a letter and she was on the phone five minutes after she'd opened it (makes sobbing sounds).”*

(Carol)

Participants perceived that discussing factors which contributed to their distress (e.g. suffering from a physical illness) with family members was potentially burdensome due to the family members’ emotional reactions. Such reactions from family members seemed to reinforce participants’ determination to manage their distress on their own.

Participants sought practical support from younger family members:

*“Well my son was the main one, he'd come and take me shopping and when they were shopping I'd go with them him and his partner, he'd drop her off then come back with me and he'd get it all out and pack it all away.”*

(Helen)

Some participants described that their children were a means to access supermarkets; this helped participants to fulfil their basic need of having food to eat.

Instead of seeking support from family members, most participants valued socialising with their friends as they often had similar problems, as reported by Diane:

*“I always feel better having been out and had a good gossip, usually we start off with all our ailments then we have a good laugh and get on with it, oh yes, having a laugh is very important, you feel better with your friends because we all suffer from one thing or another.”*

(Diane)

Community groups helped participants to retain friendships which was important as participants sought social support from friends to self-manage their distress.

### Help-seeking from healthcare services

Stigma impacted some participants’ decisions to seek help from healthcare services. Participants who had not consulted a GP when experiencing distress discussed the negative perceptions associated with mental health problems:

*“Hm, yeah, I think, well with depression and that, I don’t want to be seen as having stuff like that, do I? Its frowned upon and you shut up and shut shop and you find people say ‘no that doesn’t happen to me or in my family’.”*

(Elizabeth)

Some participants held stigmatised attitudes towards mental health problems and did not wish to be labelled as having such problems, this perhaps prevented them from consulting a GP.

Within the 12 months prior to being interviewed, five participants described consulting a GP for their mood problems. These participants described taking the decision to consult a GP due to persistent experiences of distress:

*Interviewer: “What do you think about going to the doctors [about] mental health problems?”*

*Diane: “Yeah, I tried to do it on my own until I realised I was getting churned up and I thought, I need some help.”*

Diane reported reaching a point where she could not manage her distress alone as this was not alleviating her mood problems. Persistence of mood problems was a major contributing factor for those participants who consulted a GP. Furthermore, by reporting “*I need some help*”, Diane is gaining a sense of control over her mood problems by consulting a GP.

When asked if they had sought help from healthcare services when experiencing distress within the 12 months prior to the interview, thirteen participants disclosed that they had not consulted a GP. Reasons for this included a lack in continuity of care:

*“I don't bother with the GP if I'm honest at all, having the same GP is important and seeing a different one all the time well that stops me from saying I need something, seeing a familiar face would be important.”*

(Kathleen)

Some participants described how seeing the same GP helped them to disclose symptoms of distress. Participants’ reported relationship with a GP affected their decision to seek help from healthcare services. Participants who had received a label of a mental health problem described existing positive relationships with GPs:

*“My doctors are very good they are yeah, so I feel as though I can speak to them and I've seen how they are with my husband when they've come out to him when he's been really poorly.”*

(Helen)

Participants who took the decision to seek help from healthcare services when experiencing distress reported that they could discuss their problems with a GP.

### Perceptions of treatments offered in primary care

Those participants who had consulted a GP were offered antidepressants. Owen reported that the offer of medication was made too quickly by the GP, before understanding his difficulties:

*“Essentially until they’ve looked at the problem, how can you diagnose a problem and give medication quite frankly? Well it is erm it is a stop-gap.”*

(Owen)

Owen is suggesting that he wanted his mood problem to be understood by a GP before a treatment was suggested. The five participants who consulted their GP each reported being offered medication but this treatment was not acceptable and participants preferred to act by self-managing their mood:

*Frances: “My doctor, well he offered me, erm, Valium I think, but I will not-not-not take tablets for it, no way.”*

*Interviewer: “What would you rather do to manage your mood, other than take medication?”*

*Frances: “Erm, well … er, doing my garden, I love my pots or, er, seeing people, coming to these groups actually helps a lot.”*

Rather than taking medication for mood problems, participants suggested that pursuing independent activities, or social contact with other people, would be a more acceptable solution.

Two participants, Owen and Diane, also reported being offered ‘talking therapies’ by a GP and referred to this form of treatment as “*counselling*”. Both participants who had been given the opportunity to participate in ‘talking therapies’ reported that they did not attend due to holding stigmatised attitudes towards counselling. Both Diane and Own described counselling as stigmatising:

*“If a doctor says ‘I want you to go counselling’ that is pretty much like saying you're going doo-lally and we're going to stick you in a strait-jacket and stick you in a cubicle.”*

(Owen)

Participants who did, and did not, seek help from healthcare services when experiencing distress suggested that GPs should direct older people with mood problems to third sector services:

*Interviewer: “What do you think doctors should do for distressed older people?”*

*Stephen: “Hm, well, er … maybe sending them to [community] groups like this, yeah, so they can get out and meet people and have a joke and a laugh, and maybe a piece of cake if it is somebody’s birthday (laughs) yeah, these groups are wonderful.”*

If GPs sign-posted older adults to third sector services, such as community groups, this would permit distressed older people to have social contact with other individuals.

### Visual representations of the analysis

**Fig. 1:**
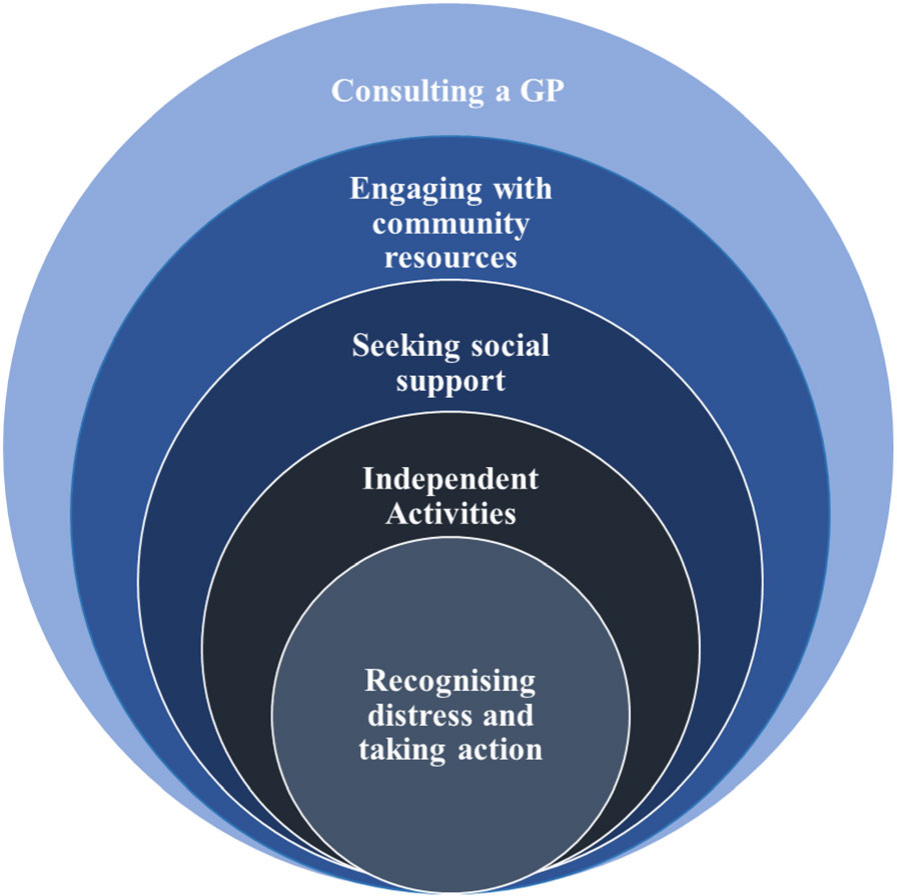
Self-management strategies

The concentric circle represented in Fig. 1 identifies the management strategies participants used when distressed.

**Fig. 2:**
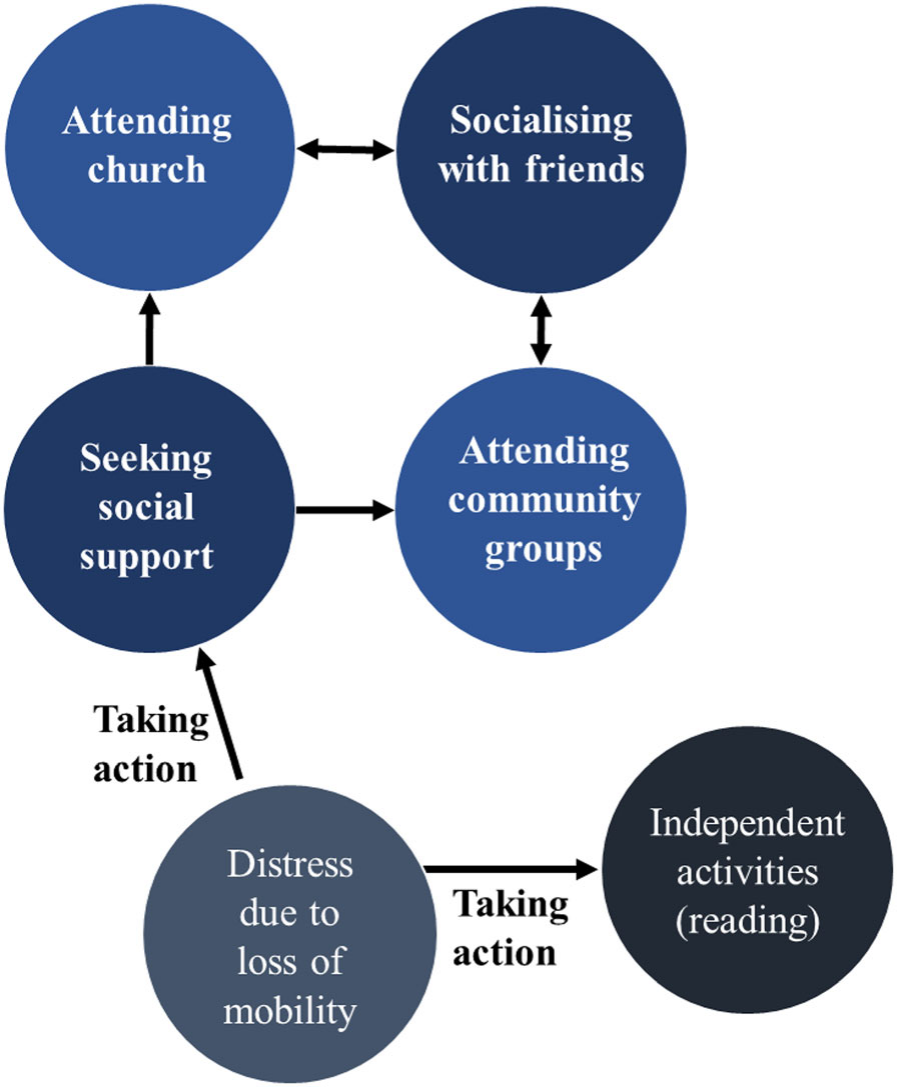
Anne’s self-management strategies

**Fig. 3:**
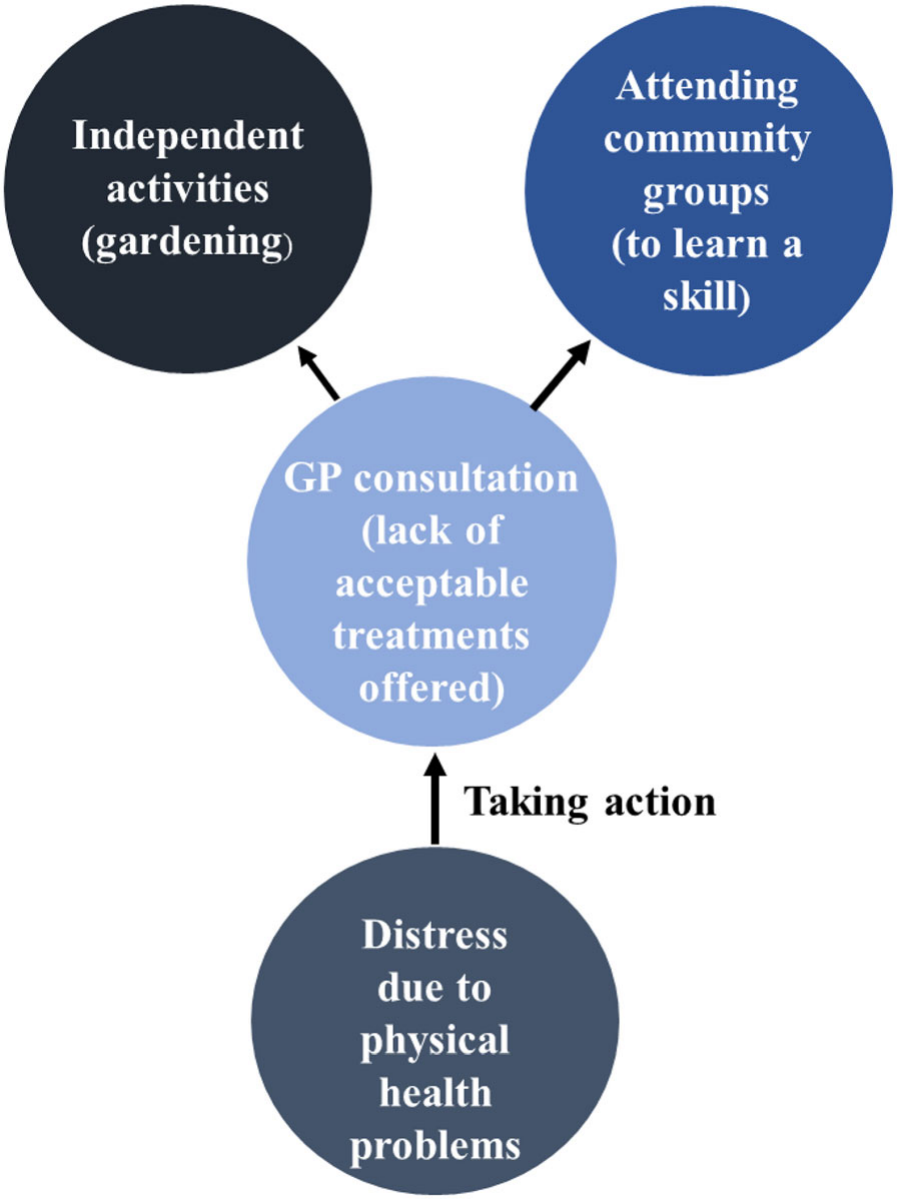
Owen’s self-management strategies

Figure 1 does not represent a linear process. Individual participants did not necessarily move from independent activities to seeking social support and then to engaging with community resources to manage their mood problems. Figures 2 and 3 illustrate how two participants managed their distress in different ways. Figure 2 is a diagram of Anne’s self-management strategies and Fig. 3 represents how Owen managed his mood problems. The colour of each self-management strategy represented in the diagrams corresponds to Fig. 1.

Figure 2 illustrates that once Anne had identified as experiencing distress, due to a loss of mobility and social contact, she took action by initiating self-management strategies. While Anne utilised reading as an independent activity that she did alone, most of the self-management strategies Anne employed revolved around seeking social support, such as socialising with friends. Seeking social support was linked to attending church and community groups as Anne sought support from individuals who also attended these community resources.

Unlike Anne, once Owen had recognised that he was experiencing distress due to physical health problems, he took action by consulting a GP who suggested a label of depression. Owen perceived the treatments offered by a GP (medication and ‘talking therapies’) as unacceptable and reported self-managing independently from healthcare services. Similar to Anne, Owen attended community groups. Owen did not report attending community groups to seek social support but to learn a skill. Owen also described gardening as an independent activity he did on his own to self-manage his feelings of distress. Figures 2 and 3 show the nuances in how different participants managed their mood problems. Each participant utilised strategies that they deemed appropriate for the management of their own mood problems.

## Discussion

This is the first study which explores how older adults understand and manage distress. Older people use a variety of terms to describe their distress, which is often caused by various forms of loss. Most participants interviewed had not sought help from healthcare services; instead they preferred to identify their own ways of self-managing their experiences of distress. Self-management strategies included: pursuing independent activities (reading, gardening, walking and practising religious beliefs), seeking social support from friends and attending community groups including church groups. Five participants had consulted a GP and described existing positive relationships with these clinicians. Other participants, who did not seek help from healthcare services, reported a lack of a relationship with one GP and did not want to be defined as having a mental health problem. The five participants who consulted a GP were offered medication but this was perceived as an unacceptable treatment and these participants preferred to self-manage their mood. Two of these participants were also offered ‘talking therapies’ but the stigma of seeking ‘counselling’ prevented them from engaging with treatment. Participants suggested that, rather than offering medication or ‘talking therapies’, the role of primary care healthcare professionals should direct older people with mood problems to third sector services (e.g. community groups).

Older adults’ use of terms such as feeling “*low*” or “*stressed”* to describe their distress has been reported in previous research [29]. To support access to treatments believed to be the appropriate solutions for distress, Geraghty et al. [11] reported that GPs were likely to label a distressed individual as ‘depressed’ because this enabled access to a broader range of treatment options such as antidepressants or ‘talking therapies’. However, the current study indicates receiving a label of a mental health problem may change how older adults view their distress as only participants who consulted a GP, and received a diagnosis of a mental health problem, used terms such as *“anxiety”* or *“depression”* to describe their mood problems. Younger adults have previously described that grief, or a series of events or stressors, contributed to their experiences of distress [29]. In our study, experiences of distress were associated with various forms of loss, these findings are similar to previous studies reporting that older people view loss as a contributing factor towards low mood or stress [30–32].

As most participants in this study preferred to self-manage their mood, this suggested that participants did not identify distress as a problem which required seeking help from healthcare services and, therefore, participants did not identify themselves as candidates for care. Candidacy describes the negotiation of eligibility for care and treatment between individuals and healthcare services [33]. Some distressed older adults may not identify themselves as candidates for care due to stigmatised attitudes and a reluctance to receive a label of a mental health problem from a healthcare professional. Past research has also found that depressed older people, who held stigmatised attitudes towards depression, did not consult healthcare services for their mood problems [34].

Persistence of mood problems was a major contributing factor for those participants who sought help from a GP. These findings suggested that if an older adult’s distress is persistent, they may present to primary care channels and, therefore, it is inappropriate for healthcare professionals to screen older adults for depression and anxiety. Within the Clinical Guideline (CG) 123, the National Institute for Health and Clinical Excellence (NICE) [35] highlighted a number of concerns about case finding for depression. Such concerns included the high rate of false-positive results identified by screening tools, the likelihood that most individuals identified by screening would have mild symptoms of depression and recover without formal interventions and the diversion of resources away from people with more serious cases of depression whose care is already often inadequate [36]. NICE (CG 123) suggests that clinicians should be alert to possible mental health problems, particularly when there has been a previous history of such conditions or when individuals are experiencing a physical health problem [35]. This study also suggested that healthcare professionals should be aware of possible mental health problems in older adults who are suffering from a form of loss.

Older people in this study described a diverse range of self-management strategies in response to distress. Depressed younger adults self-managed their mood problems by using strategies such as: seeking social support from family or friends, exercising or internet use [17–19]. As most participants preferred to self-manage their mood and valued attending community groups or church, the current findings support Kennedy et al. [37] who concluded that the self-management of physical health problems requires resources which extend beyond primary care settings. Participants who had sought help from healthcare services and who were offered medication and/ or ‘talking therapies’ perceived these treatments as insufficient solutions. Previous research has suggested that older adults did not perceive distress as a mental health problem and did not view formal treatment options as appropriate [9]; this study supports these findings. Instead, participants who consulted a GP preferred to act themselves and self-manage their mood. These findings propose that, rather than offering medication or ‘talking therapies’, the role of primary care healthcare professionals could be to direct older people with mood problems to third sector services (e.g. community groups).

If a GP believes that an older adult is experiencing distress due to a form of loss, they should be proactive in supporting these older people as this may prevent their mood problems from developing into mental health disorders, one such intervention may be the use of social prescribing. Policy initiatives have supported the integration of services between primary care and the third sector [16, 37]. In England, the NHS Long Term Plan [38] stated that nearly one million people will qualify for referral to social prescribing schemes by 2023–24. The Royal College of General Practitioners [39] also recommended that general practices introduce social prescribing initiatives as research has indicated that such initiatives can reduce symptoms of depression and anxiety [40]. This study indicated that social prescribing could provide an efficacious intervention to support older people with distress, but this relies on presentation and discussion of symptoms in primary care consultations.

### Strengths and limitations

The study’s sample was diverse in certain aspects and captured both male and female older adults who had a wide age range. The study recruited from community groups which permitted access to participants who may not have presented their distress within primary care settings. However, all participants were recruited through community groups. Distressed older adults who do not attend community groups may have had different experiences of using the Internet.

Although the sample was diverse in some respects (e.g. age, gender), all participants were White British. If the study had included older adults from different ethnic backgrounds this may have resulted in participants describing different understanding of distress and identifying other management strategies. The community groups this study recruited from charged attendees £2.50 per session: older people who could afford to attend these groups are perhaps more financially affluent than older adults who could not afford to attend. Whilst we attempted to recruit via snowball sampling, it was not fruitful and the final sample did not include any older adults who did not attend community groups. Individuals who attended community groups may have self-managed their mood somewhat differently than those who did not attend community groups.

## Conclusions

Older adults’ experiences of distress may be associated with various forms of loss, particularly the loss of physical mobility caused by physical health problems. As all participants in this study reported taking some form of action once they had recognised that they were experiencing distress, this identified that older adults are active in wanting to improve their mood. Persistence of mood problems could be a major reason for older adults to seek help from a GP. Given that contributing factors to distress may be experiences related to loss, it is important for primary care healthcare professionals to be mindful of these determinants of mental health. When older adults attend primary care services, healthcare professionals could use this opportunity to discuss psychosocial factors which could cause distress, provide information about a range of management options (which may, or may not, include medication or a referral to ‘talking therapies’), and explore older adults’ preferences and views on different management options. If an older adult does not feel that medication or ‘talking therapies’ are acceptable, the role of primary care clinicians could be to explore how a distressed older adult may self-manage their mood and consider using social prescription by sign-posting older people to local third sector services.

## Supplementary information

**Additional file 1:.** *Topic guide, *A description of the questions and areas of questioning used within the interviews

## Data Availability

The datasets generated and analysed during the current study are not publicly available due to ethical concerns, anonymised datasets are available from the corresponding author on a reasonable request.

## Abbreviations

DSM V: The Diagnostic and Statistical Manual of Mental Disorders, Fifth Addition
GP: General Practitioner
ICD-10: International Classification of Diseases, Tenth revision
NICE: National Institute for Health and Clinical Excellence
PPIE: Patient and Public Involvement and Engagement
