# Supplementary material for: How do older adults understand and manage distress? A qualitative study
Source: BMC Fam Pract. 2020 May 4;21:77. doi: 10.1186/s12875-020-01152-7 (PMC7199345; doi:10.1186/s12875-020-01152-7)
Supplement: Supplementary file 1 — Additional file 1:. *Topic guide, *A description of the questions and areas of questioning used within the interviews [file 12875_2020_1152_MOESM1_ESM.docx]

**Topic Guide**

***1) Opening***

*Clarify ethical implications, confidentiality, and consent*

*Explain the study and the aims of the research*

*Explain the purpose of the interview*

*Invite and answer any questions*

***2) Exploring the experience***

Can you tell me a little about how you came about attending this community group?

In the last few years, has there been a time that you’ve felt low or stressed?

- How did you feel at the time, can you tell me about it?

What did you do to make yourself feel better/help you cope?

What sort of help did you experience from friends or family?

- How did that make you feel?

Have you tried doing more social activities?

- How did that help (or not)?

Have you tried to do any activities on your own which helped (or not)?

Did you seek help from your GP?

- What advice was given/ treatment offered? Did it help?

Are there any other things that you tried?

- Diet, food supplements, exercise, sleep?

Have any of your friends/family ever felt low/ stressed?

- Are you aware what other people do to cope?

***5) Closing***

*Explain to the participants how the interview data will be used.*

*Ask if there is anything else that they want to say/ask.*
